# Editorial: Bridging Music Informatics With Music Cognition

**DOI:** 10.3389/fpsyg.2018.00633

**Published:** 2018-05-08

**Authors:** Naresh N. Vempala, Frank A. Russo

**Affiliations:** ^1^Psychology, Ryerson University, Toronto, ON, Canada; ^2^Nuralogix Corporation, Toronto, ON, Canada

**Keywords:** music cognition, music informatics, music emotion, computational modeling, musical preference, music representation, music segmentation

Over 30 authors contributed 15 articles toward this research topic. Collectively this body of work represents a bridge between music informatics and music cognition, covering a broad range of research topics.

We can categorize these fifteen articles into one of the following groups or a combination of them, since the groups are not mutually exclusive:

1. Research addressing problems or needs fundamental to one domain but borrowing methods, approaches, and/or insights from the other domain.
2. Research addressing problems or needs common to both domains and borrowing methods and insights from either of the two domains.
3. Research addressing problems or needs of one domain with strong implications for the other domain.

Eleven articles (i.e., 73.3%) attempt to elucidate underlying mental processes related to music. These articles may be thought of as predominantly aligned with music cognition (Baker and Müllensiefen; Barone et al.; Casey; Foubert et al.; Kim; McAdams et al.; McFee et al.; Siedenburg and Müllensiefen; Stober; van der Weij et al.; Vempala and Russo). Two articles (i.e., 13.3%) (Kaneshiro et al.; Thoret et al.) explore issues that fall mainly within the space of music informatics, while the two remaining articles (i.e., 13.3%) (Janssen et al.; Krumhansl) explore areas with research motivations relevant to both music cognition and music informatics. This cursory analysis might suggest that only limited interactions between these domains exist. With the majority of interactions biased toward music cognition, one might argue that this fragile new bridge is at risk of collapse!

However, a closer examination of the articles reveals a richer and balanced network of interactions. Of the eleven articles that are predominantly aligned with music cognition, no less than six (Barone et al.; Casey; Foubert et al.; McAdams et al.; Vempala and Russo; Siedenburg and Müllensiefen) use feature extraction methods hailing from music informatics. In other words, the dependence of these studies on music informatics should not be understated. Additionally, most of these eleven articles have moderate to strong implications for music informatics. Likewise, the two articles that fall predominantly within music informatics, have implications for music cognition.

Since all the articles present research in more than one key area within music informatics and music cognition, they may be thought of as forming dynamic clusters that may be characterized differently depending on one's vantage point. The key areas driving these clusters include but are not limited to: statistical and computational modeling, machine learning, music and emotion, musical preference and engagement, rhythm and meter perception, musical timbre and instrument identification, music similarity, music representation, structural segmentation, implied harmony, music therapy, and big data analysis.

Baker and Müllensiefen, Kim, McAdams et al., van der Weij et al., Vempala and Russo, use computational modeling as a means to explain or interpret behaviors associated with music cognition. van der Weij et al. use a probabilistic model of meter expectation to explain the effects of enculturation. But their model is generative and borrows techniques from machine learning, thus bridging into music informatics. Both McAdams et al. and Vempala and Russo explore music and emotion. While McAdams et al. examine perceived emotion based on the acoustic properties of timbre, Vempala and Russo explore higher-level emotion judgments through a classic cognitive modeling framework using machine learning methods. Baker and Müllensiefen look at how similarity in compositional structure affects salience and recognition, specifically through the use of Wagner's leitmotives. Among all the computational modeling studies, Kim's gradient frequency neural network for estimating implied harmony, is the only biologically inspired low-level computational model consisting of tonotopically tuned nonlinear oscillators.

Both Stober and Casey present findings on music representation as assessed by neural activity—a topic that intersects music cognition, music information retrieval, and cognitive neuroscience. Stober explores music imagery information retrieval through EEG recordings whereas Casey examines neural representation of music in naturalistic listening conditions through fMRI. Both studies strongly depend on machine learning and deep learning methods. Stober's work also highlights the need for sharing open datasets. Open science is a practice common to music informatics and one that is fast gaining ground in music cognition. This approach promotes collaborative research endeavors and encourages replicability of research findings.

Several studies in this topic address the importance of timbre in music. While Siedenburg and Müllensiefen focus on music similarity judgments, Thoret et al. look at timbre and the modulation power spectrum as feature sources for musical instrument identification. Thoret et al.'s work is similar to, McAdams et al. since both inspect the role of timbre in music perception. However, given the importance of automatic source recognition in music informatics, it can be argued that Thoret et al.'s work on instrument identification is more closely aligned with music informatics than music cognition.

Barone et al., Kaneshiro et al., and Janssen et al. emphasize the role of corpus analysis methods in music informatics and music cognition. Janssen et al. uses a folk music corpus to study the relationship between musical memory and melodic variation with pattern matching—research that is more traditionally aligned with music cognition but has clear implications for music informatics. Barone et al. and Kaneshiro et al. focus on the analysis of big data - an area that has become especially relevant since the advent of cloud storage and high performance computing resources. Barone et al. examine statistical regularities in music download patterns of listeners. Specifically, they look at genre and emotion preference using acoustic features. Their work serves as yet another example of research problems fundamental to music cognition using methods borrowed from music informatics.

Kaneshiro et al. also explore musical behavior of listeners at scale. They study the types of musical events within a piece of music that lead to enhanced engagement of the listener. Despite addressing issues related to perception and preference in music cognition, their work adheres more to music informatics because of its application areas comprising music discovery, multimedia search, and musical engagement.

McFee et al.'s work focuses on the analysis of musical structure, and its role in hierarchical music segmentation by annotators. They present ways to overcome limitations during the occurrence of inter-annotator disagreements because of ambiguous musical structure. Segmentation algorithms are an active area of music informatics while perception of musical structure is also integral to music cognition. As such, this research falls well within the scope of both music informatics and music cognition.

Foubert et al.'s article stands out as the only article with application in music therapy. Their research is based on the hypothesis that abnormal timing deviations during musical improvisation can be used as predictors of interpersonal relationship instability—a characteristic of borderline personality disorder. A statistical model motivated from music cognition, with rhythm and tempo-based pattern matching features borrowed from music informatics, is used to diagnose patients with borderline personality disorder.

Krumhansl's article presents the results of an extensive survey on the contexts in which people heard popular music in their lifetimes, and how they developed their preferences for music. The survey shows several interesting results about the progression of music listening across the life span of different participants. The results also provide more insights and context about different effects such as generational effects, song specific age effect, decade effect, influence of emotion on memory and preference, among others. This study has relevance for music informatics in particular, and for the music industry more generally.

Given the breadth of research occurring at the intersection of music informatics and music cognition, these 15 articles represent a small sampling. Nonetheless, through their range and diversity of topics, these articles give us a sense of the nature and scope of research at this intersection. Hence, we can safely conclude that, far from risk of collapse, the bridge between music informatics and music cognition is built on solid foundations. The diversity of interactions explored in this topic suggests that this bridge is sustainable and that it will continue to support fruitful activity for decades to come.

## Author contributions

NV was responsible for writing. FR was responsible for writing.

### Conflict of interest statement

The authors declare that the research was conducted in the absence of any commercial or financial relationships that could be construed as a potential conflict of interest.

